# The Role of EphrinB2–EphB4 Signalling Pathway in Regeneration of Inflammatory Bone Defect

**DOI:** 10.1111/jcmm.70840

**Published:** 2025-09-05

**Authors:** Lili Shen, Ning Wei, Dong Wang, Rongjing Zhou

**Affiliations:** ^1^ Department of Stomatology Liaocheng People's Hospital Liaocheng Shandong People's Republic of China

**Keywords:** bone defect, EphB4, EphrinB2, inflammatory, regeneration

## Abstract

The important role of the EphrinB2–EphB4 signalling pathway in bone remodelling has been demonstrated, while its effect on inflammatory bone defect regeneration remains poorly understood. This study was to assess the effect of EphB4–EphrinB2 signalling on inflammation‐mediated bone defect repair in murine models. The modelling method of inflammation‐mediated bone defect in mice was established by intraperitoneally injecting different concentrations of TNF‐α. Then, three randomly assigned groups were administered vehicle (PBS, control), EphrinB2 siRNA, and EphB4 siRNA into a 1.5‐mm diameter mandibular bone defect with 5 μg/kg TNF‐α intraperitoneally injected every 2 days. The gene expression of osteogenic differentiation markers Runx2, Osterix, ALP, OCN and BSP in healing tissue of the bone defect was examined by quantitative real‐time polymerase chain reaction (PCR). Runx2 and BSP protein expressions were examined by western blot, and the decalcified tissues were subjected to histological examination. Compared with the control group, the EphB4 siRNA group mice exhibited lower levels of osteogenic differentiation markers and higher levels of the osteoclastogenic marker. H&E staining, TRACP staining and bone histomorphometry showed that the bones were thinner and the number of giant osteoclasts in the EphB4 siRNA group was higher compared with the control group, whereas there were no significant differences in osteoblastic and osteoclastic differentiation between EphrinB2 siRNA mice and control mice. In conclusion, the EphrinB2–EphB4 signalling pathway plays a critical role in the inflammation‐induced bone defect repair process; selective inhibition of EphB4 using siRNA results in decreased bone formation and increased bone resorption under high inflammatory circumstances in vivo.

## 1 Introduction

Bone remodelling is not only important during bone repair after injury but is also continuously active throughout life, helping to maintain the mechanical and structural integrity of the skeleton [1, 2]. The maintenance of bone homeostasis is largely dependent upon cellular communication between osteoclasts and osteoblasts, and the coupling of bone resorption to bone formation [3]. This tight coupling is essential for the correct function and maintenance of the skeletal system, repairing microscopic skeletal damage and replacing aged bone [4]. Bone remodelling takes place at discrete bone multi‐cellular units, where the process starts with osteoclasts resorbing old bone, followed by new bone formation by osteoblasts in the resorption lacunae [5]. During the processes of remodelling, it is important to maintain a certain balance between bone resorption by osteoclasts and bone formation by osteoblasts to achieve either normal bone development or bone homeostasis [6].

The intensive researches have showed that the RANKL‐RANK‐OPG pathway plays an important role during the ‘coupling’ of osteoblast and osteoclast function in the bone remodelling process [7]. Recent studies by Zhao and colleagues also demonstrated, for the first time, the importance of EphB4 and EphrinB2 in this process [8]. The Eph molecules are a large family of receptor tyrosine kinases divided into A and B subclasses that bind to Ephrin ligands [9]. They are well known for their function as contact‐dependent repellent molecules, playing diverse roles during development and in the postnatal organism, including boundary formation, axon guidance, angiogenesis, skeletal formation and bone homeostasis [10, 11]. During bone homeostasis, reverse signalling through ephrinB2 into osteoclast precursors suppresses osteoclast differentiation by inhibiting the osteoclastogenic c‐Fos‐NFATc1 cascade, while forward signalling through EphB4 into osteoblasts enhances osteogenic differentiation. In addition, a previous report demonstrated that EphB4 was important in the in vitro osteogenic differentiation and migration of human bone marrow‐derived MSC [12]. Other studies have shown that ephrinB2 expression by osteoblasts is stimulated by parathyroid hormone receptor 1 (PTH) and PTH‐related protein (PTHrP) in a dose‐dependent manner, and that EphrinB2–EphB4 signalling promotes osteoblastic mineralization in vitro [13, 14].

In terms of important regulatory effects on skeletal formation and remodelling, EphrinB2–EphB4 has been planned to be applied to improve bone defect regeneration. However, a lot of patients who need bone regeneration procedures for bone defects caused by periodontitis, periimplantitis, or those derived from periapical pathology or post‐tumorectomy usually present with local chronic inflammation, and the role of the EphrinB2–EphB4 system in bone regeneration under this inflammatory status remains poorly understood. In this study, we investigate whether the deficiency of EphrinB2 or EphB4 could decrease osteogenic activity and enhance osteoclastogenic activity in vivo to evaluate the role of the EphrinB2–EphB4 signalling pathway in bone defect regeneration under TNF‐α‐mediated inflammatory circumstances.

## 2 Materials and Methods

### 2.1 Small Interfering RNA (siRNA) Knockdown of EphrinB2 and EphB4

EphrinB2 siRNA and EphB4 siRNA were synthesised by Life Invitrogen (Los Angeles, CA, USA) and stored at −80°C. Mouse MC3T3‐E1 cells were transfected with either EphrinB2 siRNA, EphB4 siRNA, or scramble control siRNA. Forty‐eight hours after transfection, protein and total RNA were collected. The mRNA and protein levels of EphrinB2 and EphB4 were quantified by real‐time RT‐PCR and western blot analysis separately. EphrinB2 and EphB4 antibodies (Abcam, 1:1000 dilution) were used. The sequence of scramble siRNA is CUUCACCGCAUUACUGAGCUG; the sequence of EphrinB2 siRNA is 5′‐UUTUUGGCCACUUCGGAACCC‐3′; the sequence of EphB4 siRNA is 5′‐UACUUGACCUCGUAGUCCAGC‐3′.

### 2.2 Establishment of TNF‐α‐Mediated Mandibular Defect Model in Mice

All measurements and assessments were performed by individuals blinded to all groups. This study was carried out in the specific pathogen‐free unit of the animal facility. All animals were fed sterile food and distilled water ad libitum. To minimise any potential oestrogen effects, 6 to 8 week old male C57BL/6 mice (20 ± 2 g)were used. Animals were randomly divided into 4 groups, with 6 mice in each group: (i) vehicle, PBS group; (ii) 0.5 μg/kg TNF‐α group; (iii) 3 μg/kg TNF‐α group; and (iv) 5 μg/kg TNF‐α group. TNF‐α was injected intraperitoneally every other day. Under intraperitoneal injection described as above, blood samples (6–8 draws, 150–200 μm) were taken alternately from mice's ocular angle veins at 3, 7, 10 and 14 days. Body weight of each mouse was measured respectively (20 ± 2 g at 3 days, 21 ± 2 g at 7 days, 21 ± 2 g at 10 days, 22 ± 2 g at 14 days). The concentrations of TNF‐α in the serum were determined by the enzyme linked immunosorbent assay (ELISA) kit for mouse TNF‐α (from ebioscience Systems). All procedures were done according to the manufacturer's protocols.

**FIGURE 1:**
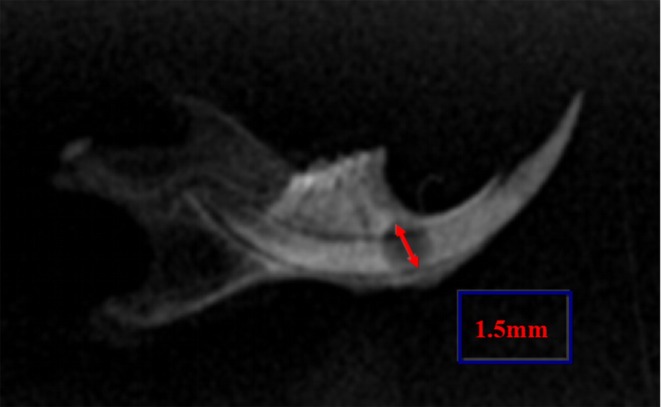
Animal surgery. A bone defect with a diameter of 1.5 mm diameter in the right mandibular was performed.

Mandibular defect model establishment: After being injected with 5 μg/kg TNF‐α, the mice were anaesthetised with an intraperitoneal injection of a mixture of Ketamine (80 mg/kg) and Xylazine (10 mg/kg), and then a bone defect with a diameter of 1.5 mm diameter in the right mandibular body was created as described previously [15]. The defect was sited below the mesial root of the lower first molar and drilled with a dental bur under continuous irrigation with phosphate‐buffered saline (PBS) (Figure 1).

### 2.3 Delivery of EphrinB2 siRNA and EphB4 siRNA Into TNF‐α‐Mediated Mandibular Defect

EphrinB2 and EphB4 siRNA were delivered to the bone defect regions using the Lipofectamine 3.0 reagent (ThermoFisher, MA, USA) according to the manufacturer's instruction. 108 mice with established mandibular defects were randomly assigned to three groups: (i) vehicle, PBS group (*n* = 36); (ii) EphrinB2 siRNA group (*n* = 36); and (iii) EphB4 siRNA group (*n* = 36). PBS, EphrinB2 siRNA and EphB4 siRNA were delivered into bone defects of randomly assigned mouse groups while intraperitoneal injection of TNF‐α every other day at a dose of 5 μg/kg in order to maintain a stable inflammatory microenvironment. Mice were killed by cervical dislocation and the mandibles were dislocated for bone biological and histomorphometric analysis.

### 2.4 Sample Collection

7, 14 and 21 days after surgery, mice were anaesthetised intraperitoneally with a mixture of Ketamine (80 mg/kg) and Xylazine (10 mg/kg). No mice died during the experiment, and the body weight of each mouse was measured at the time of sacrifice (21 ± 2 g at 7 days, 22 ± 2 g at 14 days, 23 ± 2 g at 21 days). Animals were sacrificed by cervical dislocation. Death was determined by non‐spontaneous breathing, lack of heartbeat and cold limbs, confirmed by observing cardiac and respiratory arrest for 3–5 min. Mandibular samples were isolated for further analysis.

Experimental procedures were conducted in accordance with the Institutional Animal Care and Use Committee and were approved by the Ethics Committee of Liaocheng People's Hospital (Shandong, China. Approval No. LC2021406). The primary humane endpoints used to determine when animals should be euthanized were reduced heartbeat and respiration rate. The ‘Guidelines for euthanasia of experimental animals’ was followed to minimise suffering and distress of the animals.

### 2.5 Real‐Time RT‐PCR Analysis

The overlying soft tissues were carefully removed, and the bone defect tissues were dissected and snap‐frozen in liquid nitrogen. Total RNA was extracted from the bone tissues with Trizol reagent (Invitrogen, Carlsbad, CA, USA) according to the manufacturers' instructions. RNA was quantified by measuring absorbance at 260 nm and quality assessed by the 260/280 nm absorbance ratio. 1 mg of total RNA was used for reverse transcription reaction via the PrimerScript RT Reagent Kit with gDNA Eraser (TaKaRa, Shijodori Kyoto, Japan) to synthesise cDNA. Real‐time PCR was performed using the Light Cycler Fast Start DNA Master SYBR Green I (Roche Applied Science, Penzberg, Germany). The standard PCR conditions were 10 min at 95°C, followed by 40 cycles at 95°C for 15 s, 60°C for 1 min and 72°C for 30 s. The sequences of the primers for amplification of mouse Runx2, osterix (Osx), osteocalcin (OC), ALP, EphrinB2, EphB4 and GAPDH were listed in Table 1. A melting curve was obtained for each PCR product to verify that only one amplicon was produced per PCR. Each cDNA sample was detected in experimental triplicate. The amount of mRNA was calculated for each sample based on the standard curve using the LightCycler Software 4.0 (Roche). GAPDH was used as an internal control.

**TABLE 1:** The sequences of the primers for real‐time PCR in the experiment.

Primer	Sequences	
RUNX2	Forward:	5′‐AGGTCGGTGTGAACGGATTTG‐3′
	Reverse:	5′‐TGTAGACCATGTAGTTGAGGTCA‐3′
OCN	Forward:	5′‐GCGCTCTGTCTCTCTGACCT‐3′
	Reverse:	5′‐GCCGGAGTCTGTTCACTACC‐3′
OSX	Forward:	5′‐ATGGCGTCCTCTCTGCTTG‐3′
	Reverse:	5′‐TGAAAGGTCAGCGTATGGCTT‐3′
BSP	Forward:	5′‐CAGGGAGGCAGTGACTCTTC‐3′
	Reverse:	5′‐AGTGTGGAAAGTGTGGCGTT‐3′
ALP	Forward:	5′‐CTTGCTGGTGGAAGGAGGCAGG‐3′
	Reverse:	5′‐GGAGCACAGGAAGTTGGGAC‐3′
EphrinB2	Forward:	5′‐ACGGTCCAACAAGACGTCCA‐3′
	Reverse:	5′‐GCTGTTGCCATCGGTGCTA‐3′
EphB4	Forward:	5′‐AGTGGCTTCGAGCCATCAAGA‐3′
	Reverse:	5′‐CTCCTGGCTTAGCTTGGGACTTC‐3′
GAPDH	Forward:	5′‐AGGTCGGTGTGAACGGATTTG‐3′
	Reverse:	5′‐TGTAGACCATGTAGTTGAGGTCA‐3′

### 2.6 Western Blot Analysis

Bone defect tissues were harvested and lysed, and the total protein content was determined using a BCA protein assay kit (Beyotime, Beijing, China). SDS‐PAGE and Western blot analysis were then performed using NuPAGE 4%–12% Bis‐Tris gradient gels and 0.45 μm Invitrolon polyvinylidene fluoride membranes (Invitrogen). An amount of 25 μg total protein from whole‐cell lysates was separated by sodium dodecyl sulfate‐polyacrylamide gel electrophoresis (SDS‐PAGE). Antibodies for Runx2 (Abcam, 1:1000), BSP (Santa Cruz, 1:500) and β‐actin (Santa Cruz, 1:500) were used. The secondary antibodies were horseradish peroxidase (HRP)‐linked goat‐anti rabbit IgG (Santa Cruz, CA, USA). Blots were visualised using ECL chemiluminescence reagents from Pierce Biotechnology (Rockford, IL, USA). Expression levels of Runx2 and BSP were normalised to those of β‐actin for loading differences. Images of blots were analysed using Image 4.11 program (Dahui Biotechnology, Guangzhou, China).

### 2.7 Preparation and Histological Evaluation of Decalcified Specimen

Following perfusion fixation via the intracardiac route with 4% paraformaldehyde (PFA, Sigma), the right mandibular bone was removed. Then the samples were decalcified in 10% EDTA for 3 to 4 weeks and dehydrated in an ascending series of ethanol, cleared in xylene, paraffin‐embedded and cut into 5 μm‐thick sections disto‐mesially. Sections selected from the most central areas of the bone defect were stained with haematoxylin and eosin (HE). The slides were observed with light microscopy (Olympus, Japan) and photographed using the ProgRes CapturePro software (Jenoptik Optical Systems, Germany). Three HE‐stained sections were analysed morphometrically for each sample. The new bone formation area as a percentage of the total defect area was calculated with a soft package (Image‐Pro Plus 6.0, USA).

### 2.8 Immunohistochemistry Analysis

Immunohistochemical study was performed using purified rabbit polyclonal antibody (Runx2 1:1000, OCN 1:500; Abcam, USA). Dewaxed and hydrated sections were treated with 3% H_2_O_2_ for 10 min to quench endogenous peroxidase. Then the sections were incubated with the primary antibody at 4°C overnight, washed with PBS and incubated with the anti‐rabbit secondary antibody (ZSGB‐BIO, Beijing, China) at 37°C for 30 min. Counterstaining was performed with Mayer's haematoxylin. Control sections were incubated with normal rabbit serum without the primary antibody.

### 2.9 TRACP Analysis

To quantify the number of TRACP‐stained osteoclasts in vivo, TRACP staining was performed using a leukocyte acid phosphatase staining kit according to the manufacturer's instructions (Sigma). Briefly, slides were incubated at 37°C for 30 min in AS‐BI phosphate (0.4 mg/mL) in acetate‐tartrate buffer (200 mM sodium acetate, 100 mM potassium sodium tartrate, pH 5.2). The samples were then transferred to sodium nitrite solution (1:1 v/v), in prewarmed tartrate‐acetate buffer and incubated for 30 to 60 min at 37°C until osteoclasts were bright red. Sections were then counterstained in methyl green (Sigma, St Louis, MO). Stained sections were observed under a microscope, and TRAP positive cells with three or more nuclei were counted and quantified.

### 2.10 Statistical Analysis

Statistical analysis was performed using a statistical package (SPSS 23.0, SPSS Inc., USA). All the experiments were conducted in triplicate and repeated 3 times. All values were reported as the mean ± standard deviation (SD). The normality of the data was tested using the Kolmogorov–Smirnov method, and all the data in this study are normally distributed. Statistical significance was estimated by the Student's *t* test. A *p*‐value below 0.05 was considered statistically significant.

## 3 Results

### 3.1 SiRNA Transfection Efficacy

**FIGURE 2:**
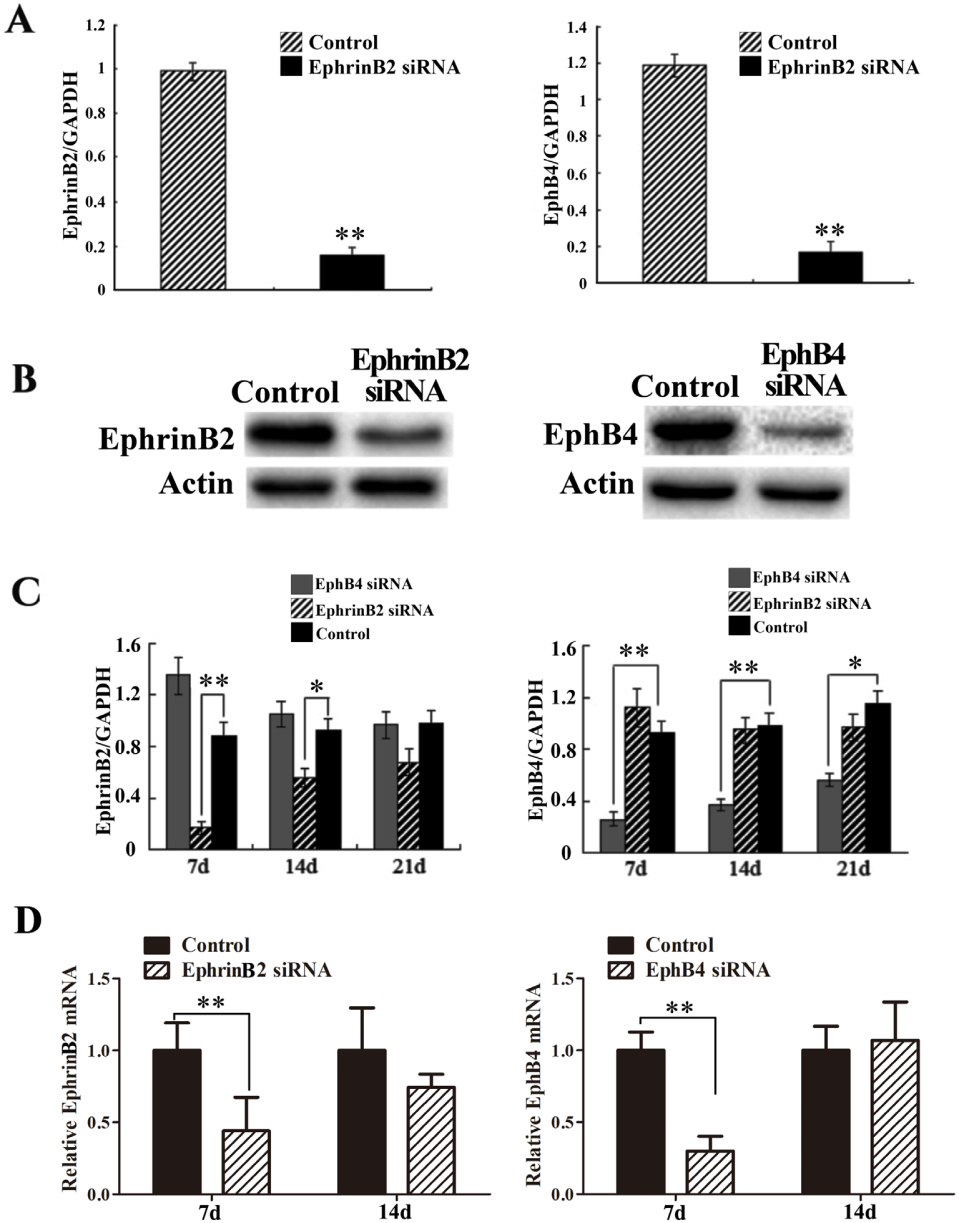
Confirmation of EphrinB2 and EphB4 deletion by PCR and western blotting. MC3T3‐e1 cells were transfected with either scrambled control siRNA or siRNA directed against EphrinB2 or EphB4 mRNA. MC3T3‐e1 cells transfected with EphrinB2 or EphB4 siRNA displayed decreased EphrinB2 or EphB4 mRNA (A) and protein levels (B). EphrinB2 or EphB4 mRNA levels in vivo (C) were consistent with those in vitro (A). (D) EphrinB2 siRNA and EphB4 mRNA siRNA were respectively injected into the bone defect area, and the expressions of EphrinB2 and EphB4 mRNA in the newly formed bone tissue were detected. Bars represent means ± SD. *n* = 6, *p < 0.05, **p < 0.01 vs. control group.

MC3T3‐e1 cells were harvested and the knockdown efficiency was evaluated with PCR and Western blotting analysis. Data showed that mRNA and protein of both EphrinB2 and EphB4 were significantly reduced 48 h after transfection compared with those of the control group (Figure 2A,B). EphrinB2 and EphB4 mRNA levels in bone tissues were also determined in order to confirm the in vivo interference result of siRNA. It was found that EphrinB2 mRNA levels were significantly decreased in the EphrinB2 siRNA group relative to those in the control group on 7 and 14 days, while EphB4 mRNA levels were markedly downregulated in the EphB4 siRNA group at all times (Figure 2C). Given that this is an in vivo study, we further verified the effect of using Lipofectamine 3.0 to deliver siRNA into the bone defect area on knocking down the target genes. The results showed that on the 7th day after transfection, the mRNA expressions of EphB4 and EphrinB2 were both significantly downregulated (Figure 2D). This suggests that in vivo transfection of siRNA using Lipofectamine 3.0 can achieve the effect of knocking down gene expression in vivo and can meet the needs of this study.

### 3.2 TNF‐α Concentration in Serum

**FIGURE 3:**
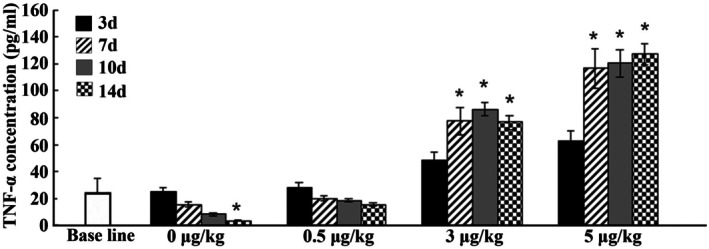
TNF‐α levels in mouse serum (pg/mL). Detection of serum inflammatory cytokine expressions at different treatment time points. *p < 0.05 vs. control group.

Serum levels for TNF‐α were obtained for all animals at different time points (Days 3, 7, 10 and 14). Cytokine levels in vehicle and 0.5 μg/kg TNF‐α treated animals decreased with time passed throughout the study. In the 3 μg/kg TNF‐α group, cytokine levels started to increase from Days 0 to 3 and reached their peak expressions at Day 7, followed by a decrease at Day 10, whereas TNF‐α concentration in the 5 μg/kg TNF‐α group increased all the time (Figure 3). Therefore, 5 μg/kg TNF‐α was used to establish inflammatory circumstances of the mandibular bone defect.

### 3.3 Osteoblast and Osteoclast Lineage Gene Expression

**FIGURE 4:**
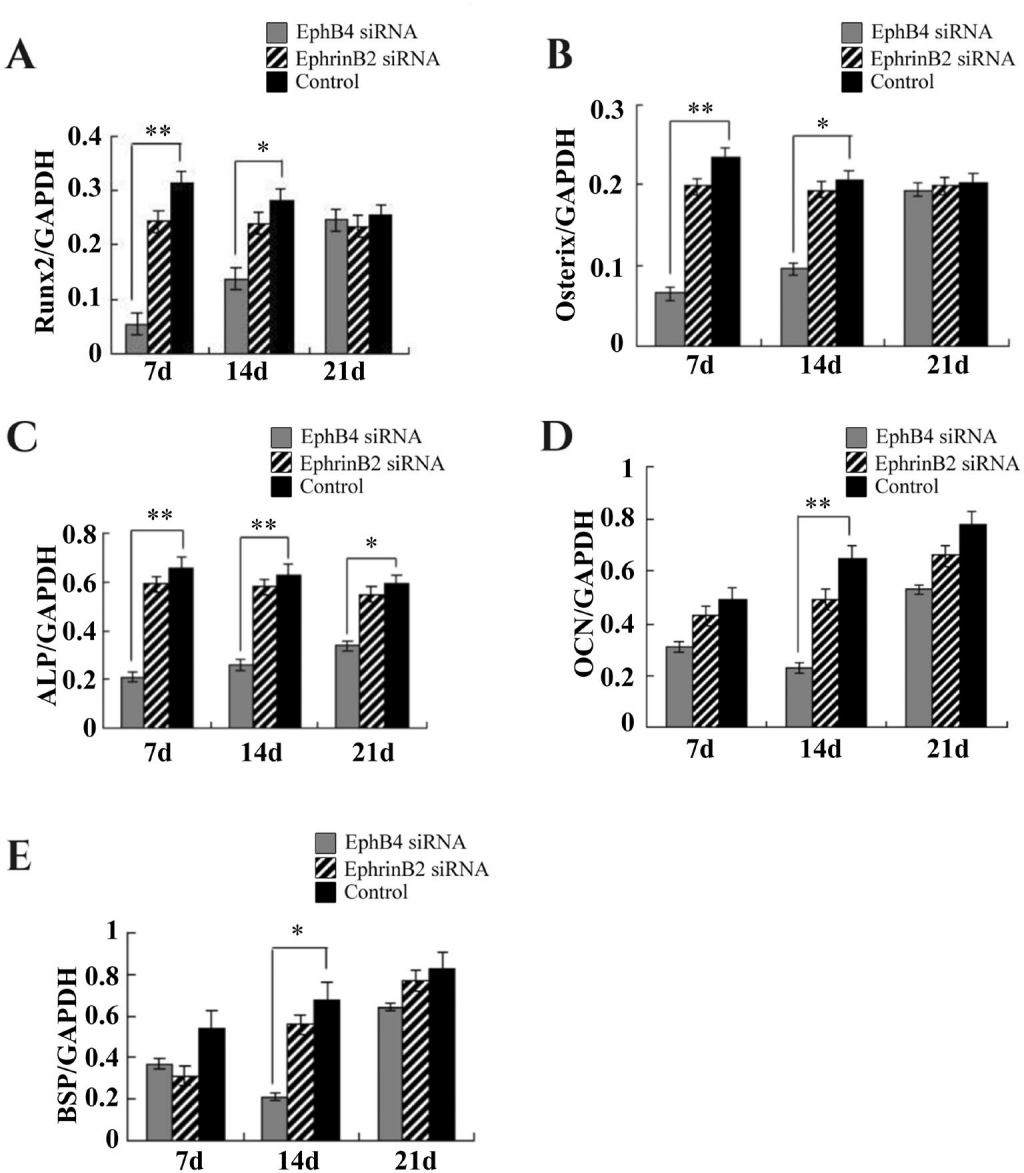
Quantitative RT‐PCR analysis of gene expression. Bone‐associated genes Runx2, Osterix, ALP, OCN, BSP, EphrinB2 and EphB4 in C57BL/6 mice were quantified by RT‐PCR. GAPDH was used as an internal reference. Data were presented as mean ± SD. These assays were repeated 3 times. *p < 0.05; **p < 0.01.

In order to explore the effect of the Ephb4–Ephrinb2 signalling pathway on bone defect repair in inflammation‐mediated circumstance, the levels of gene expression for key osteogenic and osteoclastogenic differentiation markers were monitored. We investigated RNA expression markers of Runx2, Osterix, ALP, OC and BSP using real‐time RT‐PCR on Day 7, 14, 21 for osteogenic differentiation. It was found that mRNA expressions of Runx2, Osterix (1 to 2 weeks) and ALP (1 to 3 week) were significantly lower in the EphB4 siRNA group than those in the control group (Figure 4A–C). Interestingly, there is no significant alteration of ALP, Runx2, Osterix mRNA expression in EphrinB2 siRNA group. In contrast, the mRNA expression of BSP and OCN were significantly decreased from 7‐day to 14‐day, however, with the relief of inflammation, increased on 21‐day in the EphB4 siRNA group, in which there is statistical significance compared with the control group on 14‐day (Figure 4D,E). The mRNA expression of these two factors were time‐dependently up‐regulated in EphrinB2 siRNA group, however, there is no significant difference detected between EphrinB2 siRNA group and control group which were treated with PBS.

### 3.4 Runx2 and BSP Protein Expression

**FIGURE 5:**
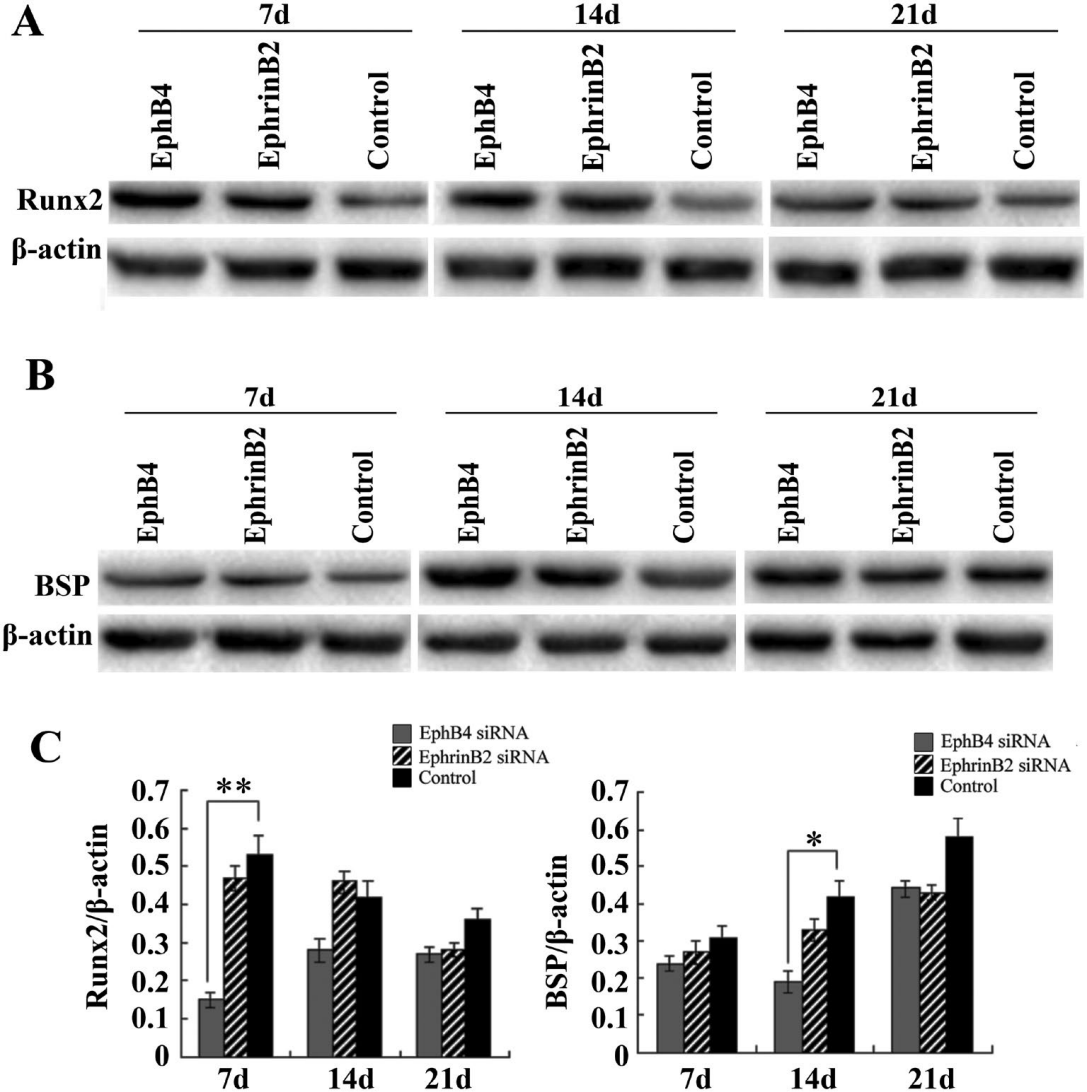
Western blotting analysis. Protein levels of Runx2 and BSP were analysed by Western Blot and quantified by Image J 1.48p. Data shown are mean ± SD. Actin serves as a loading control. These assays were repeated 3 times. *p < 0.05, **p < 0.01.

Runx2 and BSP protein expression in the bone defect was determined by western blot analysis. As shown in Figure 5, the Runx2 and OCN protein levels in the EphB4 siRNA group were significantly lower than those in the control group at 7 days and 14 days, respectively (*p* < 0.05). Interestingly, these two protein levels were lower in the EphrinB2 siRNA group compared with those in the control group; however, this difference did not reach statistical significance, which was consistent with the PCR result.

### 3.5 Histological Analysis of Bone Regeneration

**FIGURE 6:**
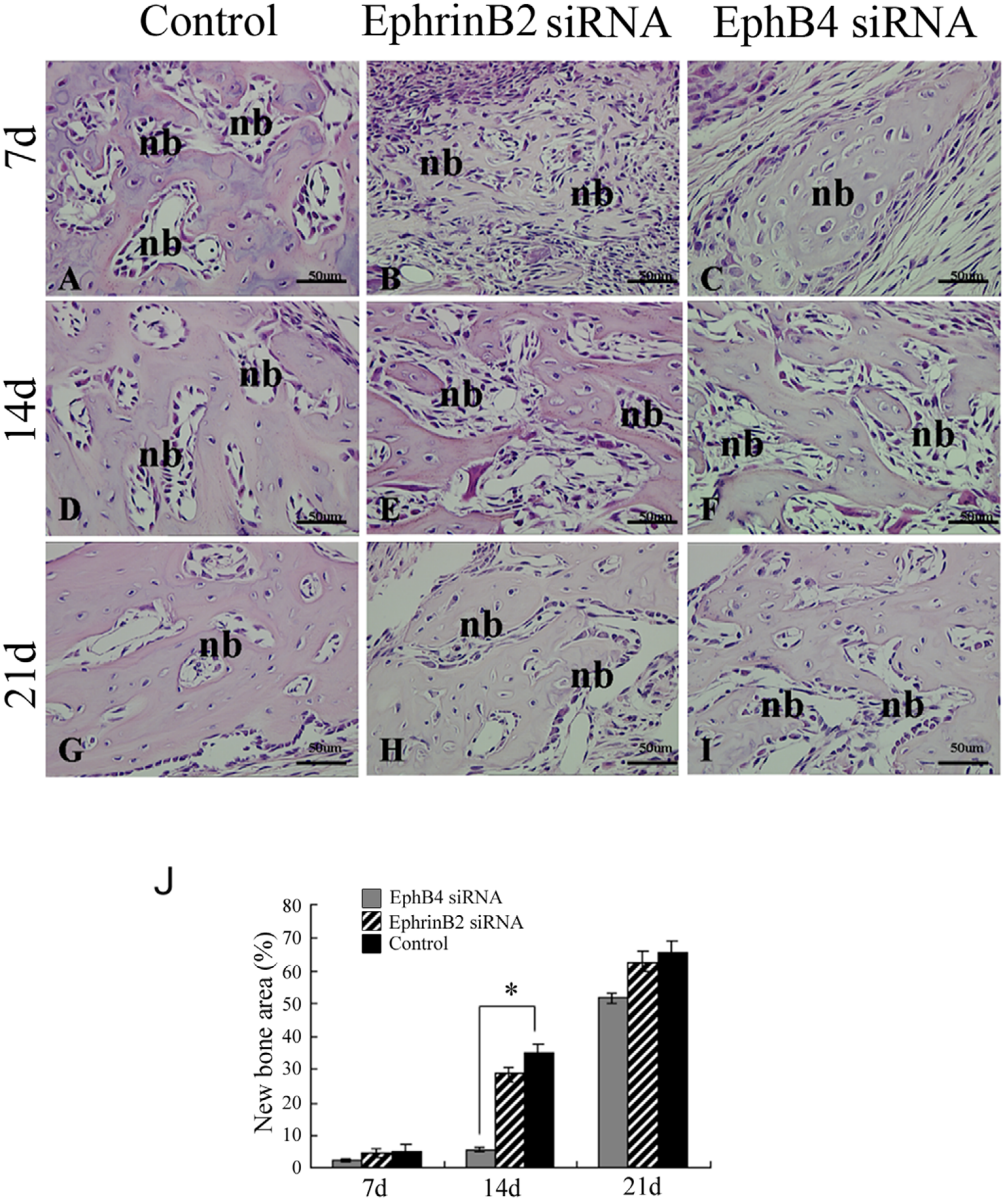
Histological observation and histomorphometric analysis within the regenerated tissue. (A–I) After paraffin‐embedded and cut into 5 μm‐thick sections disto‐mesially, the samples were subjected to HE staining and observed with light microscopy. Nb: New bones. (J) Comparison of new bone formation area among these groups. Scale bars, 50 μm. *p < 0.05, **p < 0.01.

In both experiment group and control group, newly formed bone could be observed 1 week postsurgery (Figure 6A–C). However, at this time point, no difference was detected in the percentage of newly formed bone to bone defect area among these groups (Figure 6J). Bone tissue showed a more porous pattern with less bone trabeculae, enlarged bone marrow spaces and an increased number of multinucleated giant osteoclasts. Two weeks after the surgery, the newly formed woven bone was replaced by better‐organised lamellar bone (Figure 6D–F); at this time point, the defect of the Ephb4 siRNA group showed more connective tissue and less newly formed bone than the control group, indicating the bone regeneration speed of the Ephb4 siRNA group is significantly lower than that of the control group (Figure 6F). Interestingly, the percentage of newly formed bone to bone defect area in EphrinB2 siRNA group was similar with control group (Figure 6B,F,H). Three weeks after surgery, defect areas were almost filled by newly regenerated bone in all groups (Figure 6G–I) and no significant difference was observed in these groups. Thus, the control group showed higher new bone regeneration in the peripheral area surrounding the defect compared with EphB4 siRNA groups after 2 weeks.

### 3.6 Immunohistochemical Staining Results

**FIGURE 7:**
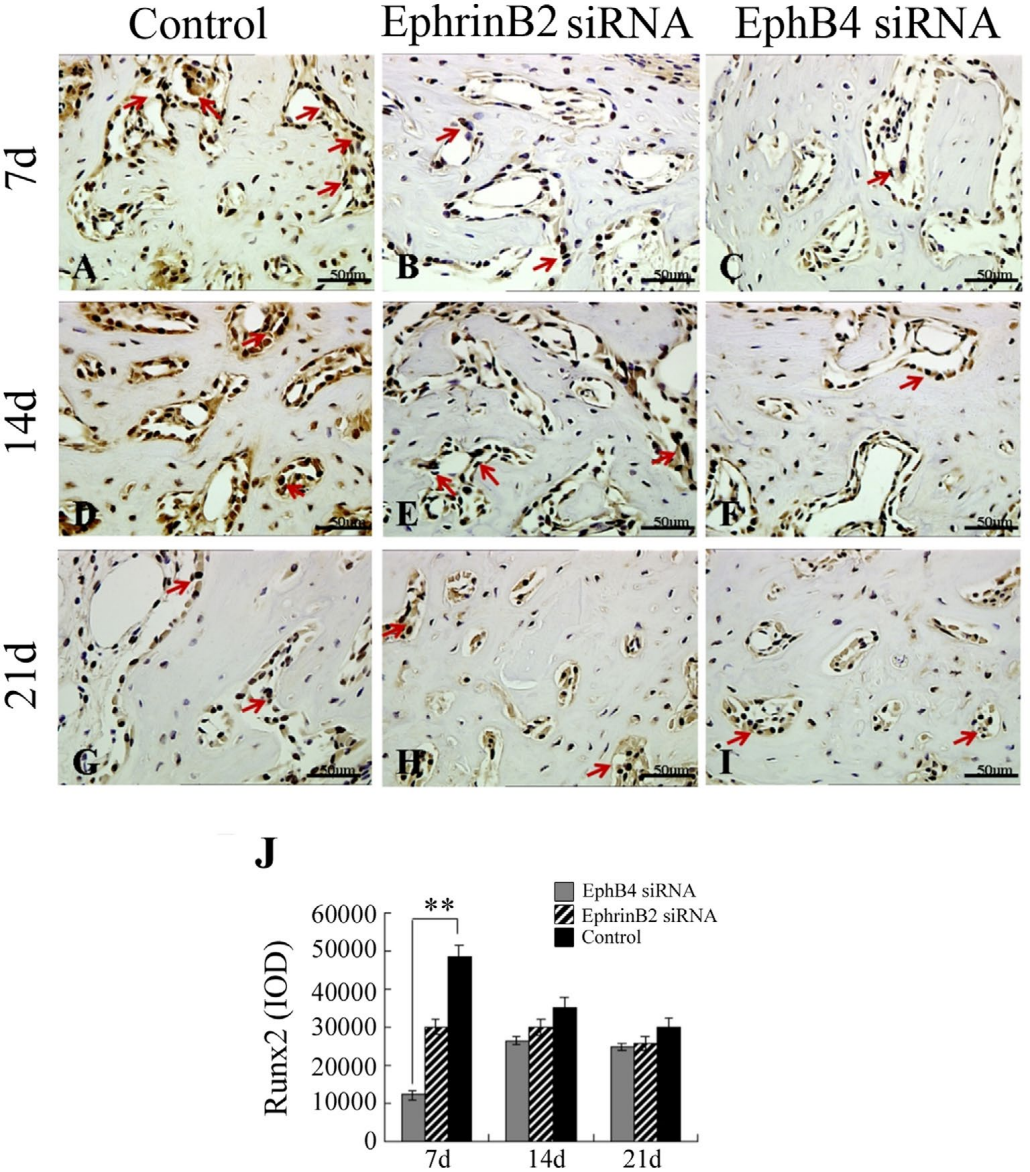
Immunohistochemistry staining of Runx2 within the regenerated tissue. (A–I) Detection of Runx2 protein expression via immunohistological staining. Different numbers of Runx2‐positive cells were stained nuclei in brown and could be found surrounding the trabecular bone in the newly formed bone area. Arrows indicate positive Runx2 staining. (J) Comparison of Runx2+ cells among these groups. Scale bars, 50 μm. **p < 0.01.

**FIGURE 8:**
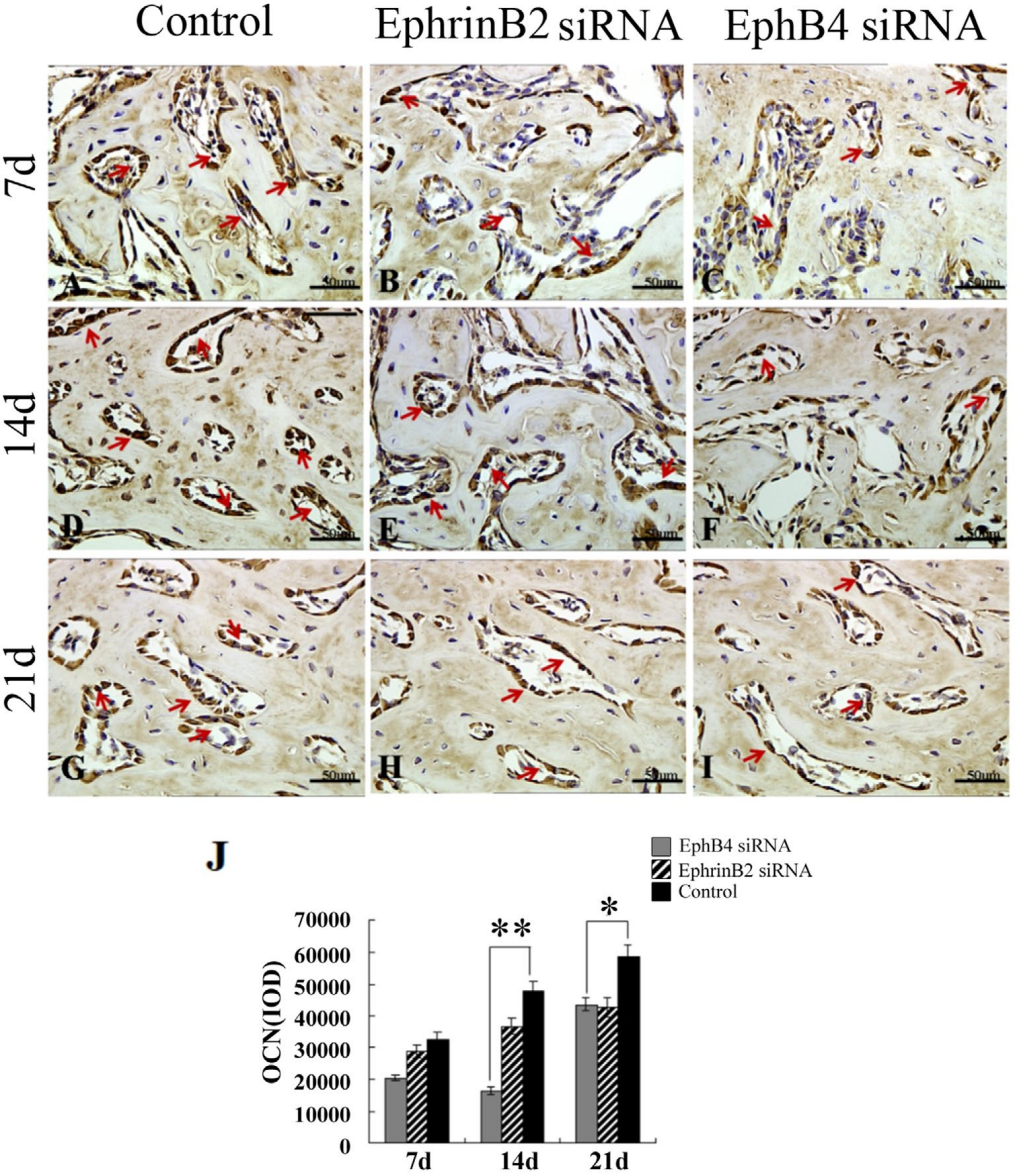
Immunohistochemistry staining of OCN within the regenerated tissue. (A–I) Detection of OCN protein expression via immunohistological staining. OCN‐positive signals could be found in the osteoblasts, osteocytes and bone matrix in the newly formed bone area. Arrows indicate positive OCN staining. (J) Comparison of OCN+ cells among these groups. Scale bars, 50 μm. *p < 0.05, ** *p* < 0.01.

IHC staining was performed to evaluate the expression of Runx2 and OCN protein in vivo, which were expressed in some osteoblasts surrounding the trabecular bone and some osteocytes buried in the trabecular bone in the newly formed bone area as a marker for osteogenic differentiation. Positive cells were counted for quantitative analysis using Image‐Pro Plus 6.0 software. We observed more intense Runx2 and OCN signals in the control group and EphrinB2 siRNA group than in the EphB4 siRNA group at every time point (Figures 7 and 8), and cell counting analysis and IOD analysis further indicated increased positive cells in the control group at every time points (Figures 7J and 8J). There were increased Runx2‐positive cells 1 week after surgery in the control group compared with the EphB4 siRNA group (Figure 7A,C) and increased OCN‐positive cells 2 weeks after surgery (Figure 8D,F). However, there was no significant difference in the number of positive cells between the EphrinB2 siRNA group and the control group at every time point (Figures 7 and 8).

### 3.7 TRAP Result

**FIGURE 9:**
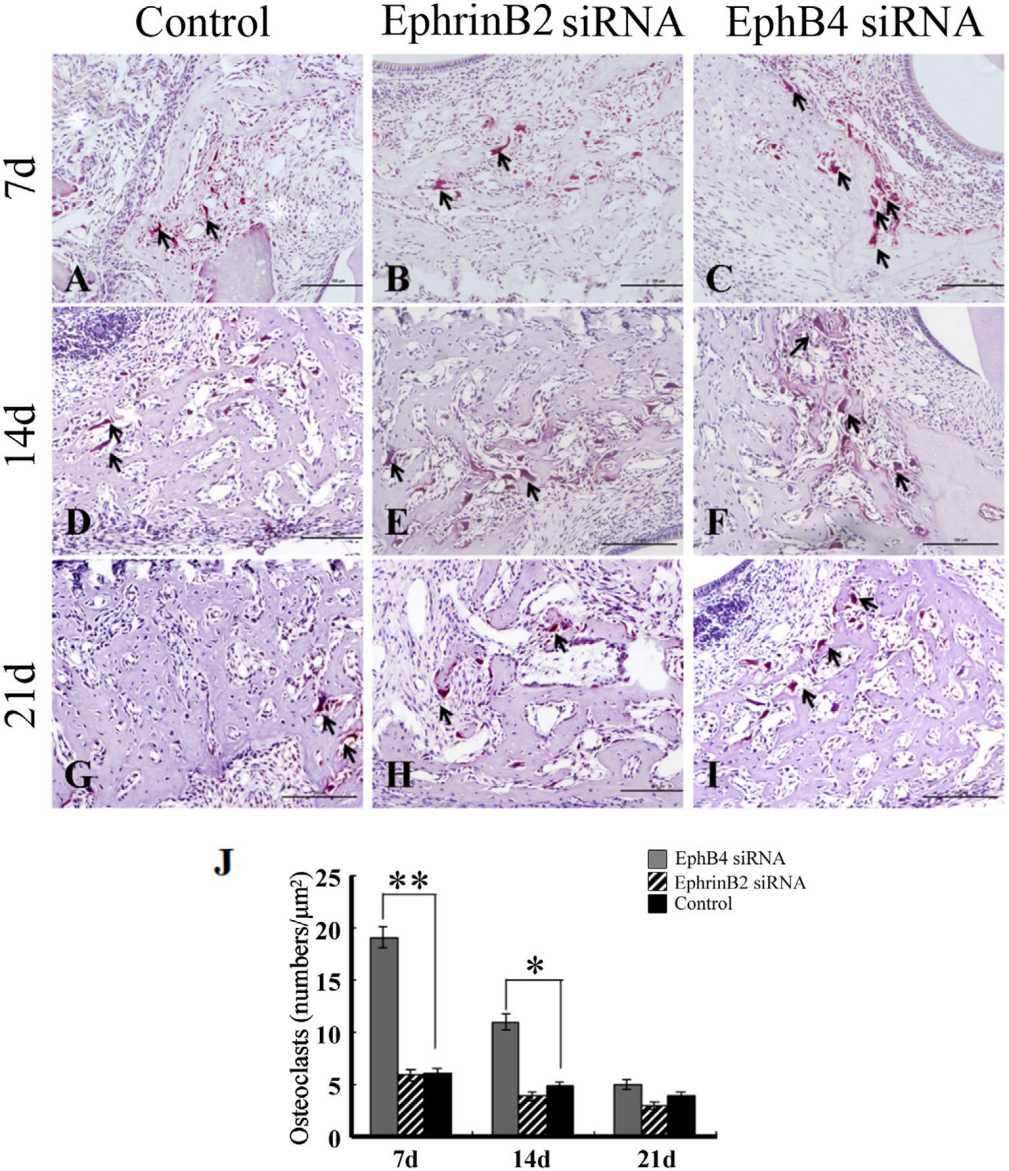
TRAP Staining analysis. Tartrate‐resistant acid phosphatase (TRAP) staining was performed on Day 7, 14 and 21, and the number of TRAP‐positive cells (Black arrow) was counted (*n* = 6 for all groups). Significantly more TRAP‐positive cells were observed in EphB4 deficient mice. Scale bars, 100 μm *p < 0.05, **p < 0.01.

To determine whether the deficiency of EphB4 or EphrinB2 affected bone resorption during the bone defect repair process, sections from bone defect areas were evaluated for TRAP‐positive cells with three or more nuclei to identify mature osteoclasts (Figure 9A–I). TRACP staining showed thinner trabecular bone and an increased number of TRACP‐positive osteoclasts in the bone defect area in the EphB4 siRNA group relative to the control group (Figure 9J). However, no significant difference in the number of TRAP‐positive multinuclear osteoclasts was detected between the EphrinB2 siRNA group and the control group.

## 4 Discussion

Cell–cell interaction mediated by EphrinB2 ligand on osteoclasts and EphB4 receptor on osteoblasts generates bidirectional anti‐osteoclastogenic and pro‐osteoblastogenic signals into respective cells and presumably facilitates the transition from bone resorption to bone formation. This makes it extremely challenging to find out the final role of EphrinB2–EphB4 signalling in osteogenic differentiation, bone formation and tissue regeneration, especially in the situation of bone disorders with persistent inflammation. Here we showed that EphB4 signalling regulated bone defect repair in the inflammation‐induced circumstance. However, unexpectedly, interference of EphrinB2 ligand did not influence bone defect repair in the same condition.

The bone defect model with persistent inflammation was just constructed on the basis of our previous study in vitro, which demonstrated that the treatment with TNF‐α at lower concentrations mainly activates the MAPK family members, which in turn display positive effects on osteogenic differentiation and enhanced expression levels of osteogenic transcription factors and bone marker genes. In contrast, higher concentrations of TNF‐α result in the activation of the NF‐κB signalling pathway, and as a result, osteogenic differentiation is inhibited. In addition, long‐term treatment with TNF‐α displays dose‐dependent inhibition in osteogenic differentiation [16]. In our present study in vivo, a mandible bone defect of 1.5 mm diameter in C57bl/6 mice was performed with intraperitoneally injection with 0, 0.5, 3 and 5 μg/kg TNF‐α, respectively [15]. To capture the progress of inflammation, serum samples were collected at Days 3, 7, 10 and 14 for levels of pro‐inflammatory cytokines TNF‐α. The result shows that only in the 5 μg/kg injection group, TNF‐α concentration increased all the time and reached a stable plasma concentration at Day 7; therefore, we choose 5 μg/kg TNF‐α as the injection concentration to maintain a stable inflammatory microenvironment.

Previous studies have demonstrated that suppressing EphB4 signalling is able to decrease the expression of several osteoblast genes involved late in osteoblast differentiation [17] and reduce the capacity of human MSC [12] and mouse bone cells [18] to form mineral in vitro, which correlated with an inhibition in the gene expression levels of osteoblastic bone‐associated genes associated with mineralization. Mice overexpressing EphB4 have increased bone formation parameters and EphB4 transgenic mice displayed a significant decrease in osteoclastic activity and an increase in osteoblastic activity [8]. These data suggest that EphB4 signalling indeed plays an important role in osteoblastic differentiation and bone regeneration under physiological conditions. However, the role of this signalling in inflammation‐induced bone defect remodelling remains poorly understood. This question is of potential significance since tissue regeneration is often needed in areas of the body where there is significant damage and hence an inflammatory reaction. Our study showed that interference of EphB4 expression by siRNA could decrease osteogenic activity, enhance osteoclastogenic activity and influence bone regeneration for TNF‐α mediated mandibular bone defect. PCR data showed decreased levels of the osteoblastic markers Runx2, Osterix, ALP, OCN and BSP mRNA in the EphB4 siRNA group relative to the control group mice. Further analysis of western blot and immunohistochemistry proved that protein expression of Runx2, BSP or OCN also significantly decreased in the EphB4 siRNA group. TRAP staining showed the significantly increased numbers of TRAP‐positive multinucleated osteoclasts within the callus in EphB4 siRNA mice compared with those in control mice. Histology demonstrated that the EphB4 siRNA group exhibited the least amount of bone regeneration when compared with vehicle control. These findings suggest that EphB4 actively participates in the bone regeneration process even under inflammatory microenvironments. One shortcoming in this point is that various indicators for osteoblastic and osteoclastic differentiation have been reported; we measured the mRNA levels of Runx2, Osterix, ALP, OC and BSP; however, in this study, we only detected the proteins of Runx2 and BSP. Of course, if more markers are detected, it will be able to better prove our conclusion.

The bidirectional anti‐osteoclastogenic and pro‐osteoblastogenic signals mediated respectively by EphrinB2 ligand on osteoclasts and EphB4 receptor on osteoblasts are considered to be a tight coupling process [8]. Therefore, interference of EphrinB2 ligand or EphB4 receptor is all expected to disturb EphrinB2–EphB4 signalling pathway and thus influence bone regeneration. Unexpectedly, knockdown of EphrinB2 by siRNA did not influence bone regeneration in this experiment. This result is inconsistent with previous studies in vitro, in which EphrinB2 over‐expression increases the osteogenic differentiation of human mesenchymal stem cells and promotes enhanced cell mediated mineralisation in a polyethyleneimine–EphrinB2 gene‐activated matrix [19, 20]. There also have been studies showed that suppressing EphrinB2 signalling is able to inhibit mineralisation and the expression of several osteoblast genes involved late in osteoblast [17], human MSCs [12] and mouse bone cells [18]. The exact causes associated with these differential effects of EphrinB2 signalling remain to be further explored. Maybe ephrinB1 or other compensatory factors may maintain functionality of EphB4 signalling pathway in the absence of EphrinB2, considering that the nature of reverse signalling of EphrinB1 and EphrinB2 is likely to be similar because the amino acid sequence of their intracellular domains is highly conserved. This implies that upon Eph binding, ephrinB1, which is also expressed in differentiating osteoclasts, signals in a manner similar to EphrinB2. Since EphB4 does not interact with EphrinB1, other Eph receptors such as EphB2 or EphB3, which are also expressed in osteoblasts, may interact with EphrinB1. Eph receptors other than EphB4 likely compensate for forward signalling mediated by EphB4 in the absence of EphrinB2, which should be determined by future pharmacological or genetic studies.

In this study, the role of EphrinB2 was detected in vitro using an osteoblast cell line. There are some literature reports indicating that EphrinB2 plays a role in osteoclast precursor cells [21]. However, there are also numerous reports on its role in osteoblasts, suggesting that the role of EphrinB2 in bone regeneration may be bidirectional [22, 23]. In our study, we only considered how EphrinB2 affects osteoblasts. In fact, there is no contradiction in EphrinB2 functioning in both osteoblasts and osteoclasts; on the contrary, this may be a mechanism through which EphrinB2 exerts its effects more efficiently. Of course, in subsequent studies, we will conduct more in‐depth research on the role and mechanism of EphrinB2 in osteoblasts.

In conclusion, the EphrinB2–EphB4 signalling pathway plays a pivotal role in the inflammation‐induced bone defect repair process. Selective inhibition of EphB4 using siRNA results in decreased bone formation and increased bone resorption, which makes possible new approaches to designing effective treatments for bone‐related diseases associated with persistent infection. Of course, this TNF‐based experimental bone defect model needs further confirmation from other animal models to strengthen its relevance for human bone disorders. In future studies, experiments are needed to examine whether EphrinB2 or EphB4 could reverse bone resorption under inflammatory circumstances and to develop EphrinB2–EphB4‐based therapeutic approaches to treat patients with metabolic bone diseases, which may not be suitable for the available antiresorptive therapies. In addition, intracellular signalling downstream of ephrins and Ephs in bone cells needs to be analysed, especially in relation to other major signals in these cells, including RANK signalling in osteoclasts and Wnt signalling in osteoblasts.

## Author Contributions

**Lili Shen:** data curation (equal), formal analysis (equal), funding acquisition (equal), investigation (equal), writing – original draft (equal). **Ning Wei:** methodology (equal), software (equal), validation (equal), writing – original draft (equal). **Dong Wang:** conceptualization (supporting), methodology (supporting), software (supporting). **Rongjing Zhou:** conceptualization (equal), funding acquisition (equal), project administration (lead), resources (equal), writing – review and editing (equal).

## Ethics Statement

The experimental procedures carried out in the present study were in accordance with the Institutional Animal Care and Use Committee and were approved by the Ethics Committee of Liaocheng People's Hospital (Shandong, China. Approval No. LC2021406).

## Conflicts of Interest

The authors declare no conflicts of interest.

## Data Availability

The datasets used and/or analyzed during the current study are available from the corresponding author on reasonable request.

## References

[1] R. Florencio‐Silva , G. R. Sasso , E. Sasso‐Cerri , et al., “Biology of Bone Tissue: Structure, Function, and Factors That Influence Bone Cells,” BioMed Research International 2015 (2015): 421746. 26247020 10.1155/2015/421746 PMC4515490

[2] I. A. Choi , A. Umemoto , M. Mizuno , and K.‐H. Park‐Min , “Bone Metabolism—An Underappreciated Player,” NPJ Metabolic Health and Disease 2 (2024): 12. 40604262 10.1038/s44324-024-00010-9 PMC12118745

[3] J. M. Kim , C. Lin , Z. Stavre , M. B. Greenblatt , and J. H. Shim , “Osteoblast‐Osteoclast Communication and Bone Homeostasis,” Cells 9, no. 9 (2020): 2073. 32927921 10.3390/cells9092073 PMC7564526

[4] A. Salhotra , H. N. Shah , B. Levi , and M. T. Longaker , “Mechanisms of Bone Development and Repair,” Nature Reviews. Molecular Cell Biology 21, no. 11 (2020): 696–711. 32901139 10.1038/s41580-020-00279-w PMC7699981

[5] L. Wang , X. You , L. Zhang , C. Zhang , and W. Zou , “Mechanical Regulation of Bone Remodeling,” Bone Research 10, no. 1 (2022): 16. 35181672 10.1038/s41413-022-00190-4 PMC8857305

[6] S. Bolamperti , I. Villa , and A. Rubinacci , “Bone Remodeling: An Operational Process Ensuring Survival and Bone Mechanical Competence,” Bone Research 10, no. 1 (2022): 48. 35851054 10.1038/s41413-022-00219-8 PMC9293977

[7] L. Wilches‐Buitrago , P. R. Viacava , F. Q. Cunha , J. C. Alves‐Filho , and S. Y. Fukada , “Fructose 1,6‐Bisphosphate Inhibits Osteoclastogenesis by Attenuating RANKL‐Induced NF‐κB/NFATc‐1,” Inflammation Research 68, no. 5 (2019): 415–421. 30927049 10.1007/s00011-019-01228-w

[8] C. Zhao , N. Irie , Y. Takada , et al., “Bidirectional EphrinB2‐EphB4 Signaling Controls Bone Homeostasis,” Cell Metabolism 4, no. 2 (2006): 111–121. 16890539 10.1016/j.cmet.2006.05.012

[9] T. K. Darling and T. J. Lamb , “Emerging Roles for Eph Receptors and Ephrin Ligands in Immunity,” Frontiers in Immunology 10 (2019): 1473. 31333644 10.3389/fimmu.2019.01473 PMC6620610

[10] A. Piffko , C. Uhl , P. Vajkoczy , M. Czabanka , and T. Broggini , “EphrinB2‐EphB4 Signaling in Neurooncological Disease,” International Journal of Molecular Sciences 23, no. 3 (2022): 1679. 35163601 10.3390/ijms23031679 PMC8836162

[11] G. Zhang , J. Brady , W. C. Liang , Y. Wu , M. Henkemeyer , and M. Yan , “EphB4 Forward Signalling Regulates Lymphatic Valve Development,” Nature Communications 6 (2015): 6625. 10.1038/ncomms7625 PMC4403310 25865237

[12] Y. Song , Y. Wu , Y. Huang , et al., “The Protective Role of Ephrin‐B2/EphB4 Signaling in Osteogenic Differentiation Under Inflammatory Environment,” Experimental Cell Research 400, no. 2 (2021): 112505. 33516666 10.1016/j.yexcr.2021.112505

[13] C. Vrahnas , M. Blank , T. A. Dite , et al., “Increased Autophagy in EphrinB2‐Deficient Osteocytes Is Associated With Elevated Secondary Mineralization and Brittle Bone,” Nature Communications 10, no. 1 (2019): 3436. 10.1038/s41467-019-11373-9 PMC6668467 31366886

[14] S. Tonna , F. M. Takyar , C. Vrahnas , et al., “EphrinB2 Signaling in Osteoblasts Promotes Bone Mineralization by Preventing Apoptosis,” FASEB Journal 28, no. 10 (2014): 4482–4496. 24982128 10.1096/fj.14-254300

[15] L. L. Shen , L. X. Zhang , L. M. Wang , et al., “Disturbed Expression of EphB4, but Not EphrinB2, Inhibited Bone Regeneration in an In Vivo Inflammatory Microenvironment,” Mediators of Inflammation 2016 (2016): 6430407. 28077917 10.1155/2016/6430407 PMC5203910

[16] H. Huang , N. Zhao , X. Xu , et al., “Dose‐Specific Effects of Tumor Necrosis Factor Alpha on Osteogenic Differentiation of Mesenchymal Stem Cells,” Cell Proliferation 44, no. 5 (2011): 420–427. 21951285 10.1111/j.1365-2184.2011.00769.x PMC6495272

[17] Y. Zhang , C. Yang , S. Ge , L. Wang , J. Zhang , and P. Yang , “EphB4/TNFR2/ERK/MAPK Signaling Pathway Comprises a Signaling Axis to Mediate the Positive Effect of TNF‐α on Osteogenic Differentiation,” BMC Molecular and Cell Biology 21, no. 1 (2020): 29. 32299362 10.1186/s12860-020-00273-2 PMC7164363

[18] F. M. Takyar , S. Tonna , P. W. Ho , et al., “EphrinB2/EphB4 Inhibition in the Osteoblast Lineage Modifies the Anabolic Response to Parathyroid Hormone,” Journal of Bone and Mineral Research 28, no. 4 (2013): 912–925. 23165727 10.1002/jbmr.1820

[19] E. G. Tierney , K. McSorley , C. L. Hastings , et al., “High Levels of EphrinB2 Over‐Expression Increases the Osteogenic Differentiation of Human Mesenchymal Stem Cells and Promotes Enhanced Cell Mediated Mineralisation in a Polyethyleneimine‐EphrinB2 Gene‐Activated Matrix,” Journal of Controlled Release 165, no. 3 (2013): 173–182. 23201622 10.1016/j.jconrel.2012.11.013

[20] W. Wang , C. Yuan , T. Geng , et al., “EphrinB2 Overexpression Enhances Osteogenic Differentiation of Dental Pulp Stem Cells Partially Through EphrinB2‐Mediated Reverse Signaling,” Stem Cell Research & Therapy 11, no. 1 (2020): 40. 31996240 10.1186/s13287-019-1540-2 PMC6990579

[21] K. Wang , Y. Kou , X. Rong , et al., “ED‐71 Improves Bone Mass in Ovariectomized Rats by Inhibiting Osteoclastogenesis Through EphrinB2‐EphB4‐RANKL/OPG Axis,” Drug Design, Development and Therapy 18 (2024): 1515–1528. 38716369 10.2147/DDDT.S454116 PMC11076049

[22] H. Yu , X. Wei , H. Jiang , H. Qi , Y. Zhang , and M. Hu , “Tensile Force Promotes Osteogenic Differentiation via EphrinB2‐EphB4 Signaling Pathway in Orthodontic Tooth Movement,” BMC Oral Health 25, no. 1 (2025): 118. 39844202 10.1186/s12903-025-05491-8 PMC11755856

[23] B. C. Heng , S. Wang , T. Gong , J. Xu , C. Yuan , and C. Zhang , “EphrinB2 Signaling Enhances Osteogenic/Odontogenic Differentiation of Human Dental Pulp Stem Cells,” Archives of Oral Biology 87 (2018): 62–71. 29272761 10.1016/j.archoralbio.2017.12.014

